# Antibody-Mediated Rejection in Heart Transplantation: Case Presentation with a Review of Current International Guidelines

**DOI:** 10.1155/2011/351950

**Published:** 2011-12-06

**Authors:** Octavio E. Pajaro, Dawn E. Jaroszewski, Robert L. Scott, Anantharam V. Kalya, Henry D. Tazelaar, Francisco A. Arabia

**Affiliations:** ^1^Department of Surgery, Division of Cardiothoracic Surgery, Mayo Clinic Arizona, 5777 E. Mayo Boulevard, Phoenix, AZ 85054, USA; ^2^Department of Medicine, Division of Cardiology, Mayo Clinic Arizona, 5777 E. Mayo Boulevard, Phoenix, AZ 85054, USA; ^3^Department of Laboratory Medicine and Pathology, Mayo Clinic Arizona, 5777 E. Mayo Boulevard, Phoenix, AZ 85054, USA

## Abstract

Antibody-mediated rejection (AMR) (humoral rejection) of cardiac allografts remains difficult to diagnose and treat. Interest in AMR of cardiac allografts has increased over the last decade as it has become apparent that untreated humoral rejection threatens graft and patient survival. An international and multidisciplinary consensus group has formulated guidelines for the diagnosis and treatment of AMR and established that identification of circulating or donor-specific antibodies is not required and that asymptomatic AMR, that is, biopsy-proven AMR without cardiac dysfunction is a real entity with worsened prognosis. Strict criteria for the diagnosis of cardiac AMR have not been firmly established, although the diagnosis relies heavily on tissue pathological findings. Therapy remains largely empirical. We review an unfortunate experience with one of our patients and summarize recommended criteria for the diagnosis of AMR and potential treatment schemes with a focus on current limitations and the need for future research and innovation.

## 1. Background

Humoral rejection is now clearly established to be a major threat to graft and patient survival after cardiac transplantation. Unfortunately, our diagnosis and treatment of cardiac allograft dysfunction has revolved mainly around understanding the cellular response and our insight into the recognition of antibody-mediated rejection (AMR) and consequently our ability to treat AMR has lagged. Humoral rejection of cardiac allografts differs from cellular rejection by targeting endothelial cells leading to the production of a capillary vasculopathy and by the infiltration of neutrophils and macrophages rather than T cells. Pathological diagnosis involves providing evidence of endothelial injury and antibody and complement deposition [1]. Thus, the diagnosis is heavily dependent on tissue biopsy confirmation. We present a recent case of ours with fatal AMR that was diagnosed postmortem and not detected by surveillance or clinically directed biopsies. Furthermore, our patient developed no detectable circulating or donor-specific antibodies. Diagnostic and treatment recommendations for AMR are reviewed. We outline the difficulties and complexity of this devastating cause of morbidity and mortality in cardiac transplantation.

## 2. Patient Case

A 24-year-old woman with a complex medical history that included idiopathic thrombocytopenic purpura requiring splenectomy and recent postpartum acute respiratory distress syndrome requiring five days of ventilatory support was transferred to our facility from a local hospital at three months postpartum in severe cardiogenic shock. She was found to have nonischemic cardiomyopathy which required emergent biventricular paracorporeal ventricular assist devices (Thoratec CentriMag, Thoratec Corp., Pleasanton, Calif, USA). After stabilization and recovery, she was listed as a UNOS status 1A (ABO, A−) for heart transplantation without detectable panel reactive antibodies (PRAs). Approximately two months subsequent to VAD placement, she underwent orthotopic heart transplantation with an HLA-compatible cadaveric heart (ABO, A+). She had a persistent postoperative coagulopathy requiring transfusion of multiple packed red blood cells, fresh frozen plasma, and platelets and eventual return to the operating room for washout and re-exploration within 24 hours of transplantation. Her retrospective cross-match was negative, and subsequent tests for donor-specific antibodies and anti-HLA circulating antibodies were negative using the Luminex solid-phase assays. She was tested for circulating antibodies multiple times throughout her care and during her final admission.

Her transplant course was complicated by multiple complications but most notably for recurrent severe infections mandating reduction in her immunosuppressive regimen. Her infections included severe genital wart outbreaks of the perineum requiring nine separate intraoperative fulgurations, as well as debridement of recurring perivulvar and peri-anal abscesses and fistula, CMV viremia and recurrent *C. difficile *diarrhea requiring oral vancomycin. Her medical complications included renal insufficiency, severe bifrontal chronic headaches requiring change in immunosuppression from tacrolimus to cyclosporine, recurrent supraventricular tachyarrhythmias with ablation therapy, antiphospholipid antibody syndrome requiring chronic warfarin anticoagulation and a thrombotic cerebrovascular accident without residual neurologic sequelae.

The patient's immunosuppressive regimen included tacrolimus, mycophenolate mofetil (MMF), and corticosteroids during the initial transplant period. Her steroids were weaned during the first three months and discontinued completely in the maintenance period because of the recurrent infections described above and her preoperative history of splenectomy. By fourteen months postoperatively, she was converted to cyclosporine. Her headaches resolved after the change in calcineurin inhibitor. For the last twelve months of her life, she was treated with two drug therapy, cyclosporin and MMF. Her cyclosporin dose was intermittently decreased when she presented with clinical infections. Her MMF dose was decreased during infections or episodes of neutropenia from 1000 mg twice a day to 500 mg twice a day but maintained at 1000 mg twice a day during her final 12 months. In her final week, her cyclosporine was reduced from 150 mg twice a day to 75 mg twice a day. Her trough cyclosporine levels were maintained at around 200 ng/mL in her final months but during periods of infection were allowed to drop below 100 ng/mL.

Despite the required changes in her immunosuppressive regimen, the patient had only one documented episode of mild cellular rejection diagnosed at biopsy in the first perioperative month. She gradually developed evidence for mild diastolic dysfunction over the first two years following transplantation. Based on left and right heart catheterization results and echocardiographic and biopsy results, there was concern that she was developing mild constrictive or restrictive physiology. Her ejection fraction gradually decreased from 70% in the first few months after transplantation to 50% at 29 months after-transplant. She showed no evidence of significant mitral or tricuspid regurgitation. Heart biopsies taken throughout her course and during the months that she developed cardiac dysfunction showed no evidence for cellular or humoral rejection. The evaluations included staining for C4d (complement product) and CD68 (a macrophage specific marker). She underwent two heart biopsies in her last nine months with the last being 4 months prior to her death. Again, both of these biopsies were negative for cellular and humoral rejection in the setting of worsening ventricular function. Left heart catheterization showed no evidence of coronary vasculopathy.

**Figure 1:**
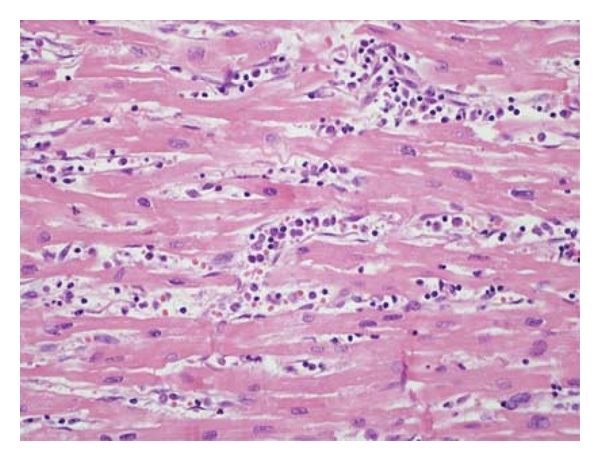
Antibody-mediated rejection characterized by endothelial cell swelling and numerous macrophages filling vascular spaces.

**Figure 2:**
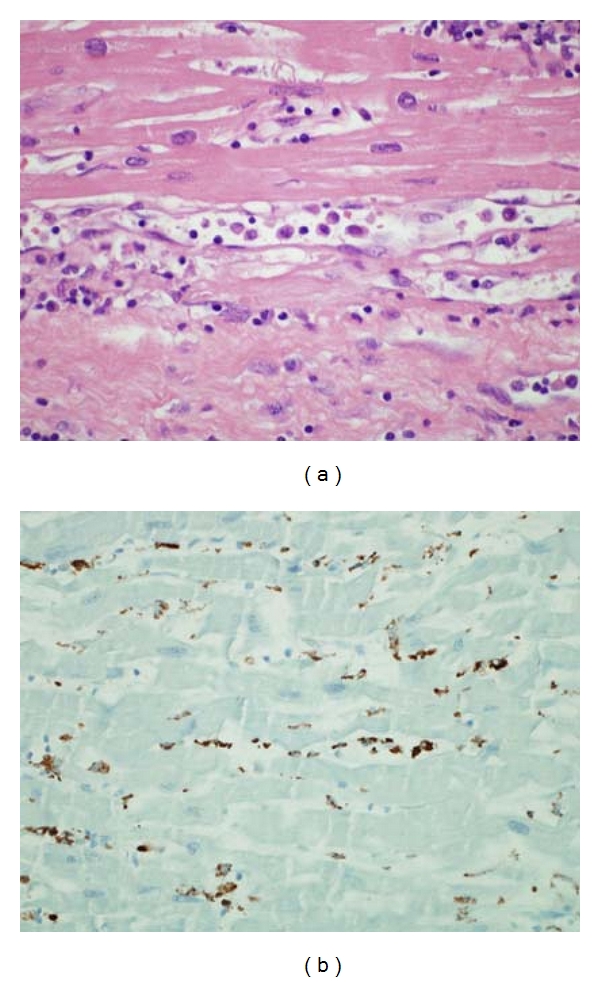
(a) Antibody-mediated rejection, high power. Longitudinal section of capillary with mild endothelial cell swelling and macrophages accumulating in lumen. (b) Immunoperoxidase staining with CD68 highlights the macrophages.

**Figure 3:**
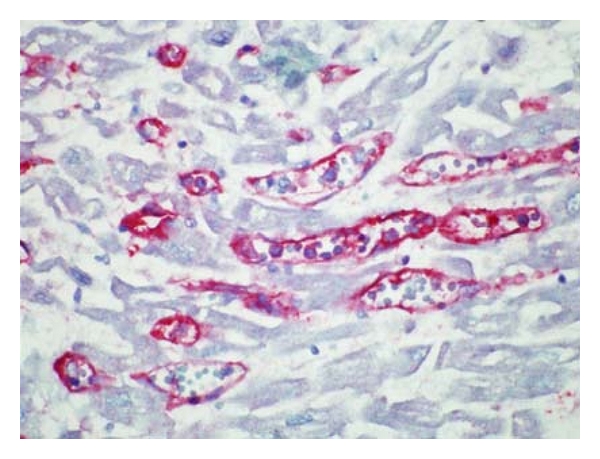
Antibody-mediated rejection, high power. C4d decorates endothelial cells in this immunoperoxidase-stained slide.

At approximately 29 months after transplantation, the patient was admitted for diarrhea, dehydration, emesis, and acute renal insufficiency. She responded well to rehydration; however, she experienced an episode of hypoxemia leading to complete heart block from which she was easily resuscitated but required intubation. A urine screen was positive for both temazepam and alprazolam, which the patient had been prescribed as an outpatient and the cause of her arrest was attributed to hypoxemia secondary to hypoventilation and sedation. She was subsequently noted to have right upper and left lower extremity swelling, and ultrasound revealed superficial venous thrombosis, despite continued Coumadin anticoagulation. A chest scan to rule out pulmonary embolism was performed and was negative. She became febrile and was placed on broad-spectrum antibiotics. On the day of her death, she developed recurrent third-degree heart block and ventricular tachycardia. Despite aggressive resuscitation efforts, she died that evening. An autopsy was performed, and although the initial gross evaluation failed to show the cause of her graft failure, histologic and pathologic myocardial sections eventually revealed severe but patchy cellular and antibody-mediated rejection. (Figures 1, 2(a), 2(b), and 3 show the pathologic findings of antibody-mediated rejection in the case described here.)

## 3. Review and Discussion

### 3.1. Overview

The case presented here, although truly unfortunate in its outcome and unusual in its presentation because of the insidious recurrent infections, illustrates the complex difficulties encountered in the management of heart transplant recipients. First, we have no adequate measure of the degree of immunosuppression. This patient's history of recurrent and life-threatening infections obliged us to alter our immune therapy. However, we did so without the existence of an adequate measure of the “immune state.” While we did measure drug levels and peripheral blood cell counts, we did not have a set of parameters or reliable markers which could tell us in a prognostic and diagnostic manner this patient's risk of rejection or infection as we altered her immune therapy. This limitation exists in the management of all cardiac recipients. Ultimately our patient developed fatal cellular and humoral rejection. Second, this lack of a noninvasive predictive clinical measure forces clinicians to take an expectant approach, that is, an approach referred to as “heightened rejection surveillance.” This approach, which involves increasing the number and frequency of biopsies is essentially a “wait and see” approach. Thus, as clinicians, we are obligated to manage immunosuppression by reacting to the most recent adverse event. Third, endomyocardial biopsies “the gold standard” for detecting rejection, can fail to detect both cellular and humoral rejection. This is a direct result of the fact that these pathophysiologic rejection processes are not homogenous and, thus, the heart biopsy in addition to its direct risk of injury to the heart and the patient is inherently limited by the fact that it is a random sample. Interobserver variability has also been shown to be a limitation of the cardiac biopsy [2]. The case presented here illustrates clearly the clinical consequences of the heterogeneous, patchy nature of both cellular and antibody-mediated rejection. Thus, a significant risk of a false-negative biopsy result exists. Fourth, the clinician on suspicion alone is often forced to treat suspected rejection events empirically with direct pathologic confirmation lacking. We were reluctant to do this for this patient without tissue confirmation because of the incessant and recurrent life-threatening infections in her posttransplant course. Equally complicating is the ambiguity of defining and treating antibody-mediated rejection, a diagnosis dependent on biopsy proof with or without detectable circulating or donor-specific HLA antibodies. In this patient's case, the lack of pathologic confirmation and the lack of detectable circulating antibodies made it difficult to treat her empirically for AMR.

### 3.2. Gene-Expression Profiling

The goal of developing a prognostic and diagnostic test that is a measure of the patient's “immune state,” that is minimally invasive and that could ultimately be used to diminish the need for endomyocardial biopsies has been approached aggressively by studies supported by XDx, Inc. (Expression Diagnostics) in two major clinical studies. The first study, entitled CARGO (Cardiac Allograft Rejection and Gene Observation), was published in 2006 and led to the development of a proprietary gene expression test called the Allomap [2, 3]. The hypothesis as stated by the company in performing the CARGO study was that a gene-expression pattern in the peripheral blood mononuclear cells could discriminate between patients with no rejection and those with moderate or severe cellular rejection. By identifying low-risk patients for rejection, a subset of patients could be monitored without the need for invasive biopsies. They assumed that gene expression profiling in the peripheral blood would predict rejection at the organ level. The CARGO study identified 11 classifier genes involved in important immune activation pathways including T-cell activation, T-cell migration and macrophage activation, hematopoiesis, and steroid responsiveness. The initial findings of the CARGO study (Deng et al. [3]) were corroborated by the first clinical experience published by Starling et al. [4]. The Allomap was developed and consists of the 11 classifier genes and 9 control genes. The assay has a sensitivity of 84.6% at identifying the low-risk patient and a negative predictive value of 99.6%. The CARGO investigators note, however, that the historically labeled “gold standard,” the endomyocardial biopsy, suffers from significant interobserver variability and is, thus, an imperfect tool to use for gene discovery. Previous studies have confirmed the difficulty of using the endomyocardial biopsy as a gold standard and documented inter-observer variability [2].

Allomap is now available on the market but has been received with significant skepticism and the lack of enough clinical validation has made it difficult to get insurance companies to pay for it. To address this, a multidisciplinary and multiinstitutional study group was formed that included 13 US cardiac transplant centers [5]. The study group set up a trial called Invasive Monitoring Attenuation through Gene Expression (IMAGE) whose goal was to validate the use of gene expression profiling for rejection surveillance. The results were published last year, and the investigators concluded that there was noninferiority of gene expression profiling with endomyocardial biopsy in rejection surveillance after cardiac transplant. The study was met with criticism for several reasons among which was the fact that most patients were enrolled more than a year after transplantation at a time when cardiac biopsy is of unproven benefit [6]. The highest risk of acute cellular rejection for cardiac allografts occurs in the first six months with a gradually tapering risk throughout the first year. Furthermore, both the gene expression profiling and the cardiac biopsies were poor at predicting the endpoints of the study. While somewhat disappointing at predicting rejection, the gene-expression profiling did prove non-inferiority to an imperfect “gold-standard” and the approach is highly attractive because of the potential for both prognostic and diagnostic information and the minimal risk peripheral blood gene expression profiling poses.

### 3.3. Diagnosing Humoral Rejection

Antibody-mediated rejection still poses unanswered and complex questions. The role of antibody-mediated rejection in acute and chronic cardiac allograft dysfunction is now firmly established [7–9]. However, the topic has been steeped in controversy in heart transplantation stemming from an inability for many years to come to a consensus on the clinical and pathologic diagnosis and on the appropriate treatment [10–12]. In part, the difficulty in defining this process arises from the still emerging understanding of the cellular and molecular mechanisms involved. The lack of a consensus on these issues has made it difficult to assess the true incidence of cardiac humoral rejection. Over the last five years, the International Society of Heart and Lung Transplantation has published two consensus papers on cardiac antibody-mediated rejection [1, 13]. The most recent paper has made some progress in defining the clinical entity and produced a tentative outline of a pathological diagnosis. The multidisciplinary and international committee made important steps forward in agreeing on several clinical aspects of cardiac humoral rejection. Cardiac dysfunction, circulating antibodies, and donor-specific antibodies are no longer required to make the diagnosis. In 2005, cardiac dysfunction was considered to be absolutely required. Thus, the committee has acknowledged the possibility of asymptomatic antibody-mediated rejection as published by Wu et al. [14] and that the antibodies responsible may simply not be detectable in the peripheral blood. However, a pathologic diagnosis is still required. While the committee did not provide a grading scale at the moment, it did agree that the pathologic diagnoses had to include evidence of endothelial “activation” with evidence of intravascular macrophages, neutrophil infiltration, and injury to the capillaries. The committee felt that only capillary vessel analysis should be included in the pathologic evaluation. Immunofluorescence should include evidence of complement activation by staining for C3d or C4d and staining for HLA to evaluate injury to the endothelial capillaries. CD68 should be used to assess for macrophage accumulation. In the case presented here, we were unable to detect cellular or humoral rejection until postmortem despite obtaining biopsies during her decline in cardiac function. On her final admission, biopsies were not obtained because of her presentation with diarrhea and dehydration and the lack of change in her echocardiogram from one obtained four months earlier with a negative biopsy.

We may have been aided by the use of two potential approaches. One approach was published in a recent study by Kobashigawa et al. [15] which showed a correlation in between low ATP levels (<200 ng/mL) in peripheral blood leukocytes and the incidence of infection in heart transplant recipients using the ImmuKnow assay manufactured by Cylex Inc. (Columbia, Md, USA). In this study, rejection and infection episodes were analyzed in 337 patients who had also undergone ImmuKnow assays. The patients were from 2 weeks to 10 years after-transplant and had undergone 1187 ImmuKnow assays. Assays from patients with an infection or rejection event within one month prior to the assay were not included (323 assays from 41 patients). All patients were treated with a three-drug regimen (tacrolimus, mycophenolate, and corticosteroids) without induction. Assay scores were correlated with infection and rejection events that were within one month after the ImmuKnow assay. While the study is limited by its size, a significant correlation was found between a low assay score and an infection event occurring within one month of the measurement (187 ± 126 ng ATP/mL in 38 infections versus 280 ± 126 ng ATP/mL in 18 patients in steady state). The authors point out that the study was too small to discriminate between patients with impending rejection and those in steady state. Intriguingly, the highest assay scores were obtained in 3 of the 8 patients with rejection. These patients had a score of 491 ± 121 ng ATP/mL (significantly higher than those from patients in steady state) and exhibited antibody-mediated rejection.

Another approach that may aid us in the future is the possibility for the potential biopsy diagnosis of antibody-mediated rejection involving the analysis of endothelial cell gene expression profiling [1, 16, 17]. Specific gene expression patterns may be indicative of endothelial injury and may be detectable even without detecting complement products. An approach utilizing the peripheral blood leukocyte assay (ImmuKnow) and the gene expression profile obtained at biopsy may have helped us in earlier diagnosis of antibody-mediated rejection and steered us away from focusing primarily on an infectious etiology.

### 3.4. Treating Humoral Rejection

Therapy in AMR should aim toward improving graft dysfunction, prevention of long-term complications such as coronary allograft vasculopathy (CAV), and improving graft survival. As mentioned above, the International Society of Heart and Lung Transplantation 2005 guidelines included allograft dysfunction as required criteria in the definition of AMR [13]. The recent recommendations suggesting that asymptomatic patients with AMR, with no demonstrable graft dysfunction, have an increased risk of coronary vasculopathy and death [1, 14] and that neither circulating nor donor-specific antibodies are required for the diagnosis raise immediate practical therapeutic concerns. First, the clinical impact of currently available therapy for the asymptomatic patients without graft dysfunction would be hard to measure and thus demonstrating and monitoring the benefits would be difficult in the short term. Also, therapies directed at reducing circulating and donor-specific antibodies would be impossible to evaluate in patients with no detectable antibodies other than with repeat biopsies. Again, the sampling bias of the biopsy would potentially lead to false conclusions about efficacy of treatment. Further risk stratification of these patients based on other parameters is required. Nonetheless, the current recommendations revolve around minimizing antibody-mediated myocardial injury. The general therapeutic options include removing circulating antibodies, reducing activation and differentiation of B lymphocytes, minimizing the activation of complement, and suppression of T-cell activation [1].

Plasmapheresis is very effective at rapid removal of circulating antibodies. Two most frequently used techniques are plasma exchange method and double-filtration plasmapheresis [1]. Both forms of plasmapheresis are nonselective and do not specifically remove immunoglobulins. Immunoadsorption plasmapheresis is a more specific modality of antibody removal using an adsorbent membrane but the expense and availability of the adsorbing membrane limits its utility [1]. The duration of treatment can vary from days to weeks. A rebound effect producing an increase in circulating antibodies following plasmapheresis treatment requires additional therapy either with intravenous immunoglobulin (IVIG), calcineurin inhibitors (CNI), or rituximab (an anti-CD20 monoclonal antibody targeting developing and mature B cells but not plasma cells). All three types of plasmapheresis carry the risk of volume depletion, infection, exposure to fresh frozen plasma or the adsorption membrane [1]. Intravenous Immunoglobulin (IVIG) has been generally used to treat allosensitization in patients with elevated panel reactive antibodies prior to undergoing cardiac transplant [18]. Successful IVIG therapy in treating renal and heart transplant AMR has been reported [19–21]. In one study, five patients who met criteria for AMR (C4d, C3d deposition on biopsy and with concomitant graft dysfunction) were treated successfully with IVIG and plasmapheresis and two patients with donor specific antibodies were given rituximab in combination with plasmapheresis [22].

The efficacy of antithymocyte globulin (ATG) in treating AMR in heart transplantation is unclear, even though it has been used to treat AMR in renal transplantation [23]. Calcineurin inhibitors and antiproliferative agents such as mycophenolate mofetil (MMF) and sirolimus, which are the mainstay immunosuppressive therapy in heart transplant, have not been directly studied in treating AMR in cardiac allograft recipients. Tacrolimus therapy in combination with sirolimus or MMF may be superior in treating both cellular and humoral rejection compared to cyclosporine/MMF-based therapy [24].

Rituximab, which is known to inhibit activation and maturation of B cells, has been used to treat AMR in heart transplant patients. Rituximab, given as a weekly dose for up to four doses and administered in combination with plasmapheresis, IVIG, and steroids, has been used successfully to treat AMR and to reduce PRA in pre-heart transplant [25–27]. The utility of bortezomib, a proteosome inhibitor used for treating multiple myeloma, while used in renal transplant patients with AMR, is limited to desensitization protocols for reducing panel reactive antibodies (PRA) in patients with AMR, when used in combination with IVIG, rituximab, and plasmapheresis [28, 29].

Complement activation is an important factor by which circulating alloantibodies induce graft injury. Eculizamab is a humanized monoclonal antibody which prevents activation of complement component by binding to C5 and thus preventing generation of C5a and the membrane attack complex. Eculizamab has been approved for treatment of paroxysmal nocturnal hemoglobinuria in the USA. Early experience has shown success in treating AMR in renal transplant patients [30, 31].

Total lymphoid irradiation has been used to treat rejection in heart transplant patients but appears to increase the risk of hematologic malignancies [32]. Photopheresis has been demonstrated by some to be successful in treating recurrent rejection in cardiac transplant recipients with severe hemodynamically compromising cellular rejection. This modality appears to be better tolerated with fewer side effects [33]. Its utility in treating AMR in heart transplant has not been established.

No treatment strategy in cardiac transplantation has consistently proven to be successful in treating AMR. Tacrolimus-based therapy, in combination with MMF and steroids, appears to be effective in preventing AMR. Future trials may provide more insight into the utility of newer therapies with rituximab, bortezomib, and anticomplement antibodies.

## 4. Summary

In its simplest formulation, the clinical efforts of post-heart transplant management involve three basic goals: prevent rejection, minimize infection, and minimize the adverse side effects of the immunosuppressive therapy. Currently, diagnosis is highly dependent upon pathological tissue evidence. While patients can be treated empirically, the hope is that advances in molecular biology including transcriptome and proteomic analyses will help improve our ability to manage patients following heart transplantation. New diagnostic modalities will include refinement of molecular testing to provide diagnostic and prognostic markers for rejection and infection. Newer treatment modalities for antibody-mediated rejection will target antibody production and complement activation. It is likely that a percentage of patients with unclear etiology of graft dysfunction may be suffering from undiagnosed cellular and/or humoral rejection because of the heterogeneous nature of the rejection process.
